# Modulating Hole Transfer from CdSe Quantum Dots by Manipulating the Surface Ligand Density

**DOI:** 10.1021/acs.nanolett.5c01323

**Published:** 2025-05-23

**Authors:** Chari Y. M. Peter, Chayan Carmenate Rodríguez, Hannah N. Gorski, Elizabeth O. Phinney, Todd D. Krauss, Ellen M. Matson

**Affiliations:** † Department of Chemistry, University of Rochester, Rochester, New York 14627, United States; ‡ Institute of Optics, University of Rochester, Rochester, New York 14627, United States

**Keywords:** hole transfer, partial ligand stripping, colloidal nanocrystal, quantum dot, surface chemistry

## Abstract

The structure and density of surface capping ligands in cadmium chalcogenide quantum dots (QDs) are important considerations for controlling the efficiency of charge separation via the transfer of electrons or holes to molecular acceptors. Here we show how the manipulation of the surface ligand density of oleic acid-capped cadmium selenide (CdSe) QDs impacts the efficiency of hole transfer (HT) to polyoxovanadate alkoxides. Meerwein’s salt is used as a ligand-stripping agent, providing opportunities to quantitatively manipulate the ligand density at the surface of the nanocrystal, as evidenced by ^1^H NMR spectroscopy. Time-resolved photoluminescence and transient absorption spectroscopies reveal that the extent of HT is quantitatively related to increased surface accessibility. Collectively, these results show that the reduction of surface ligand density can be used to tune the extent of interactions of molecular acceptors with QDs, providing a route to control charge-transfer processes relevant to improving the efficiency of QDs as photosensitizers.

## Graphical abstract

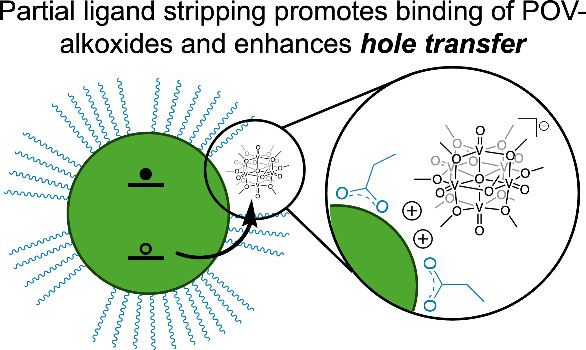

Colloidal semiconductor nanocrystal quantum dots (QDs) are emerging as versatile materials for photocatalysis, especially as applied to solar fuel production and organic chemical transformations.^1–9^ QDs absorb light strongly,^10–12^ are resistant to photochemical degredation,^13,14^ are able to perform multielectron charge transfer reactions,^15^ and have size-tunable redox properties.^16–18^ Collectively, these traits contribute to their excellent performance as photocatalysts or photosensitizers in photochemical reactions.^19^ However, the efficient extraction of photoexcited holes remains a significant challenge.^20–22^ The photoexcited hole tends to be trapped on the QD surface at highly localized defect sites,^23,24^ which reduces the probability of interacting with a molecular acceptor in solution. Additionally, these localized surface trap states reduce the driving force for hole transfer (HT) reactions.

The chemical structure and density of QD surface capping ligands, including length, chemical functional groups, and orientation, are important factors for determining the efficiency of hole (and electron) transfer from QDs to molecular acceptors in solution.^25–28^ For example, a large ligand surface density provides a barrier that can impede access for the acceptor to the surface of the nanocrystal, limiting the probability of charge transfer.^29^ The electronic structure of surface ligands can also be used to delocalize QD excited states onto the ligands, which has been shown to accelerate HT dynamics.^21,30,31^ Changing the QD concentration changes the interactions between nanocrystals and their ligands in complex ways that are dependent on ligand chemical functional groups and the ligand binding affinity, which influence the efficiency of charge transfer.^32^ In addition to these factors, the additional roles that ligands play in stabilizing the colloidal nanocrystal solution make quantitative understanding of how ligands affect charge transfer difficult to achieve.^2^

One possible way to enhance access for charge acceptors to the QD surface is to remove capping ligands. Prior work has demonstrated the ability to form “bare” CdSe QDs following the addition of excess ligand-stripping reagents [e.g., nitrosonium tetrafluoroborate (NOBF_4_), fluoroboric acid (HBF_4_), and hexafluorophosphoric acid (HPF_6_)].^33–35^ However, forming bare QDs without etching the surface of the nanocrystal is difficult. One exception is trimethyloxonium tetrafluoroborate (Meerwein’s salt, MS), which has been shown to be a particularly gentle substrate for the removal of capping ligands.^33,36^ MS removes ligands via methyl cation transfer, irreversibly stripping the ligands from the QD surface in a nonacidic and nonoxidizing manner.^37^ However, stripped nanocrystals often possess a reduced colloidal stability. As such, we postulated that the addition of stoichiometric equivalents of MS to CdSe QDs would *partially* remove surface ligands, allowing for greater accessibility of molecular acceptors to the QD surface, while retaining the colloidal stability of the nanocrystal in nonpolar organic solvent. This would, in turn, render our approach a simple way to tune charge transfer efficiency.

Here, we report control over HT from CdSe QDs to a molecular polyoxovanadate alkoxide (POV-alkoxide) complex through quantitative tuning of the surface ligand density of the nanocrystal. Oleic acid (OA)-capped CdSe QDs have a tightly packed, hydrophobic ligand shell, which inhibits access of molecular acceptors to the QD surface. The addition of discrete quantities of MS to OA-capped CdSe QDs results in the irreversible removal of ligands from the surface of the nanocrystal. The addition of POV-alkoxides results in HT from the stripped QDs to the cluster, with the relative efficiency of HT dependent on the amount of ligand removed from the nanocrystal surface. Time-resolved photoluminescence (PL) spectroscopy studies are employed to determine the number of POV-alkoxide clusters (1–2) strongly interacting with the QD. The number of clusters that can strongly interact with the QDs was consistent with the fraction of available surface area for POV-alkoxide cluster–QD interactions based on the amount of ligands that were removed. Overall, these results suggest that the QD surface capping ligand density can be controlled in such a manner as to provide a facile route to tune QD-based photocatalysis.

For initial ligand stripping studies, OA-capped CdSe QDs were synthesized according to a variation of an established procedure^22^ (see the Supporting Information for details on the CdSe QD synthesis). The resultant nanocrystals were then treated with 20 equiv of MS per QD. The addition of MS to OA-capped QDs results in the liberation of ligands from the QD surface as methyl oleate,^38^ as was confirmed by ^1^H NMR spectroscopy (Figure 1). Unlike fully stripped nanocrystals, these partially stripped CdSe QDs retain their solubility in nonpolar organic solvents.

**Figure 1:**
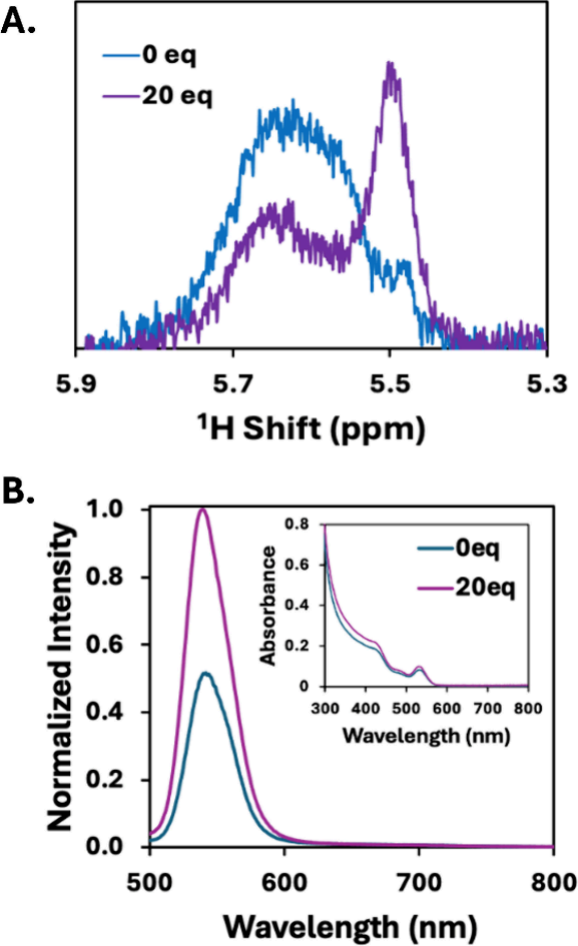
(A) ^1^H NMR spectra of OA-capped CdSe QDs with 0 (blue) and 20 (purple) equiv of MS in toluene-*d*_8_. The peaks at 5.5 and 5.6 ppm correspond to free methyl oleate and bound OA, respectively. (B) Normalized PL spectra and absorbance (inset) of OA-capped CdSe QDs with 0 (blue) and 20 (purple) equiv of MS added in dichloromethane.

It is important to assess the effect of MS on QD photophysics. QD absorption spectra show similar exciton absorbance peak positions and line widths upon the addition of 20 equiv of MS (Figure 1B, inset), suggesting that the size and shape of the QD is not significantly modified following the addition of the stripping reagent. This was confirmed through transmission electron microscopy (Figure S1), where native CdSe QDs (2.66 ± 0.30 nm) and nanocrystals treated with 20 equiv of MS (2.64 ± 0.18 nm) possess identical size distributions. Interestingly, the addition of 20 equiv of MS to CdSe QDs results in a significant increase in the PL intensity of the nanocrystal, corresponding to a PL quantum yield (QY) increase from 2.2% to 5.2% (Figure 1B). Increased QD PL QY has been previously observed when organic ligands are stripped from the surface of a nanocrystal with halide-containing reagents.^39,40^ The halides are believed to passivate defect sites on the QD surface, eliminating nonradiative recombination in surface trap states.^40^ We postulate that, following treatment of the nanocrystal with MS, the tetrafluoroborate anions (or fluoride impurities) passivate the QD surface in a similar manner.

Initial experiments performed to evaluate the ability of partially stripped QDs to transfer charge to POV-alkoxides focused on steady-state PL spectroscopy (Figure 2). Following treatment of a QD sample with 20 equiv of MS, [V_6_O_7_(OCH_3_)_12_]^1–^ (**V**_**6**_ **O**_**7**_^**1–**^) was added. Increasing PL quenching was observed as the equivalents of **V**_**6**_ **O**_**7**_^**1–**^ added to the solution were increased (0–20 equiv, Figure 2A). In contrast, the PL of the as-synthesized OA-capped CdSe QDs (i.e., no MS added to the sample) exhibited less quenching following the addition of 20 equiv of **V**_**6**_ **O**_**7**_^**1–**^ (Figures S2 and S3). Collectively, these experiments support our hypothesis that the removal of a subset of OA ligands from the surface of the nanocrystal enhances charge transfer.

**Figure 2:**
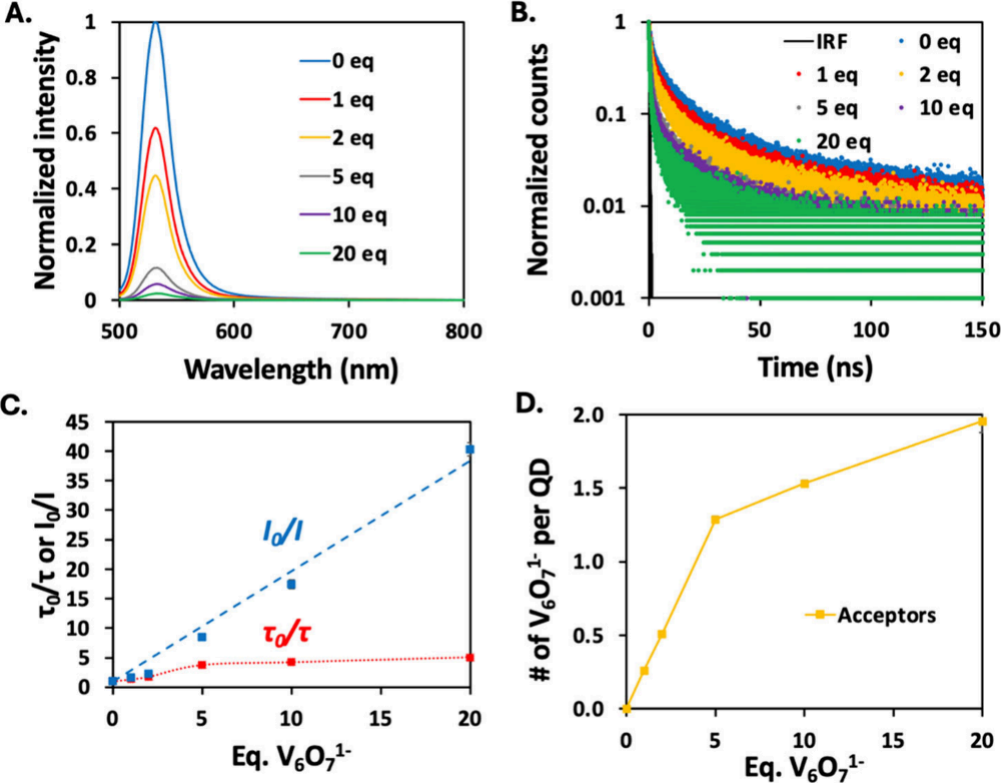
(A) Normalized PL intensity and (B) normalized PL kinetics of OA-capped CdSe QDs + 20 equiv of MS with an increasing number of **V**_**6**_ **O**_**7**_^**1–**^ equivalents. (C) Steady-state (blue) and time-resolved (red) PL quenching. The red dotted line is a guide to the eye, while the blue dashed line is a linear fit. τ_0_ is the amplitude-weighted average PL lifetime of just the QDs + MS, and τ is the lifetime with added **V**_**6**_ **O**_**7**_^**1–**^. (D) Calculated average numbers of acceptors per QD (orange solid line), which are related to the number of equivalents of **V**_**6**_ **O**_**7**_^**1–**^.

Steady-state PL quenching in OA-capped CdSe QDs can result from a combination of electron transfer, HT, and energy transfer. **V**_**6**_ **O**_**7**_^**1–**^ is capable of accepting electrons or holes from the QD (the relevant energy states are within the band gap of CdSe; Figure S4). Energy transfer between the QD and cluster as a possible source of quenching is minimal due to the limited spectral overlap between the QD donor and **V**_**6**_ **O**_**7**_^**1–**^ (Figure S5; see the Supporting Information for details).^22^ To distinguish between electron transfer and HT, transient absorption (TA) spectroscopy was performed. For CdSe QDs, TA spectroscopy primarily measures electron transfer dynamics and thus is not sensitive to HT.^41^ As shown in Figure 3, the TA bleach magnitude remained nearly constant over the first 50 ps of probe delay, regardless of the amount of added POV-alkoxide. For probe delay times much longer than is expected for HT, some modest absorption bleaching is observed (Figure S6) in the case of 20 equiv of **V**_**6**_ **O**_**7**_^**1–**^ per QD. Notably, this relatively small amount of bleaching is an order of magnitude less than what would be needed to fully account for the observed, steady-state PL quenching (Figure 2A). We have previously observed similar long-lived exciton dynamics for QDs in the presence of POV-alkoxide clusters, which was attributed to back-electron transfer from photoexcited QDs to the oxidized cluster.^22^ Collectively, these findings indicate that HT is the dominant PL quenching mechanism.

**Figure 3:**
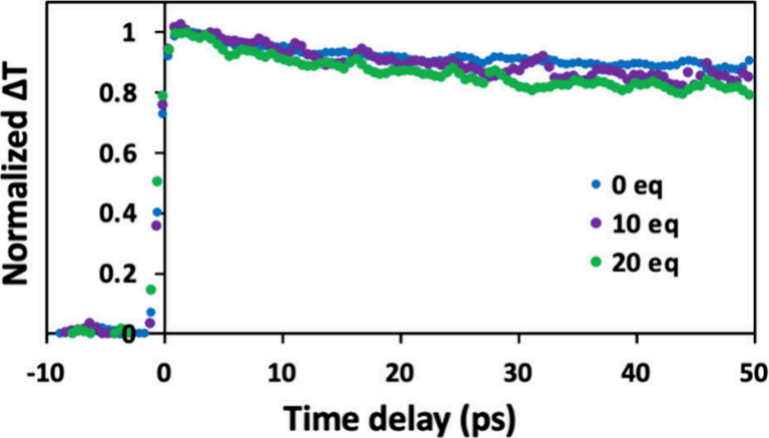
Normalized change in transmission (Δ*T*) as a function of the probe delay of the 1S_e_–1S_h3/2_ transition of stripped OA-capped CdSe QDs (20 equiv MS per QD) without **V**_**6**_ **O**_**7**_^**1–**^ present (blue) and with 10 (purple) and 20 (green) equiv of **V**_**6**_ **O**_**7**_^**1–**^.

Intrigued by our initial findings, we next explored how the extent of ligand stripping impacts the HT efficiency by adding a lower equivalent of MS to OA-capped CdSe QDs (2–20 equiv). Monitoring the alkene region of the ^1^H NMR spectra, we observe a decrease in the intensity of the broad bound OA peak at 5.6 ppm with increasing amounts of MS added. As the intensity of the bound ligand peak decreases, there is an increase in the signal associated with methyl oleate at 5.5 ppm, consistent with the stripping of OA ligands from the nanocrystal surface upon the addition of MS (Figure S7). Quantification of the bound and free ligand species was achieved via integration under the respective bound OA peak and the corresponding free methyl oleate against a ferrocene internal standard (Figure S9 and Table S1).^38,42,43^ We observe a decrease in the number of OA ligands bound to the QD and a subsequent increase of free methyl oleate in a manner that correlates to the amount of MS added.

A Stern–Volmer analysis of the steady-state PL quenching data was used to understand how changing the capping ligand density at the QD surface affects the efficiency of HT (Figure 2C). For a given amount of MS, the QD PL intensity (*I*) was plotted as a function of the increasing cluster concentration according to *I*_0_/*I* = 1 + *K*[**V**_**6**_ **O**_**7**_^**1–**^], where *K* is the Stern–Volmer quenching constant and *I*_0_ is the original PL intensity in the absence of cluster. We hypothesize that as the native ligands are stripped the amount of available QD surface increases, which enhances the probability for an interaction between the QDs and the cluster, leading to more efficient quenching of PL through HT.

Figures 2C and S11–S14 show the PL quenching efficiency as a function of added cluster for between 2 and 20 equiv of MS per QD. For low MS concentration (2 and 5 equiv per QD; Figures S11 and S12), the Stern–Volmer plot shows an initial linear increase in the *I*_0_/*I* value, which starts to show some evidence of plateauing at 5–20 equiv of cluster present. The downward curvature in the Stern–Volmer plot can be explained in terms of the existence of two populations of fluorophores, one of which is highly accessible to quenchers and the other being relatively less accessible.^44^ We hypothesize that the saturation of the quenching interaction between the clusters and the QDs is due to a small number of available binding sites at the surface of the nanocrystal upon the addition of low equivalents of MS. In other words, the relatively small amount of surface ligands that have been removed from the QD surface restricts access to the cluster. For the larger equivalents of MS to QD, the Stern–Volmer plot is linear, indicating that enough ligands have been stripped such that the entire QD is potentially available for interactions with **V**_**6**_ **O**_**7**_^**1–**^ (Figures 2C, S13, and S14).

It is notoriously difficult to assign the underlying quenching mechanisms to the steady-state PL quenching data. Thus, we next examined the PL decay dynamics of the QD under conditions identical with those for the steady-state PL quenching (see the Supporting Information for details on the PL lifetime measurements and global fitting procedure to obtain PL lifetimes). Generally, we found that the fluorescence lifetime of CdSe QDs decreases as a function of added cluster, as shown in Figure 2B for 20 equiv of MS per QD. Similar to the steady-state PL quenching data, we applied a Stern–Volmer analysis to the time-resolved PL quenching data according to *t*_0_/*t* = 1 + *k*[**V**_**6**_ **O**_**7**_^**1–**^], where *t* is the fluorescence lifetime in the presence of the cluster, *t*_0_ is the fluorescence lifetime in the absence of the cluster, and *k* is the dynamic quenching constant (Figures S11–S14).

The PL lifetime Stern–Volmer plot shows saturation behavior consistent with a heterogeneous QD–cluster population. For all concentrations of MS added, we see an overlap between the steady-state and time-resolved Stern–Volmer plots (Figures 2C and S11–S14) for lower equivalents of cluster (0–2 equiv). We interpret this behavior as arising from a freely diffusing QD-cluster population participating in dynamic quenching. From 5 to 20 equiv of cluster added per QD, there is an increase in *I*_0_/*I*, while τ_0_/*τ* remains almost constant. This behavior, which we attribute to a population of strongly associated QDs and clusters having significant PL quenching, is such that the shortened lifetime is unmeasurable (Figure 2C). A similar behavior has been observed previously for HT from tetradecylphosphonic acid (TDPA)-capped CdSe QDs following association with oxygen-deficient POV-alkoxides.^22^

We can estimate the number of molecular acceptors (**V**_**6**_ **O**_**7**_^**1–**^) interacting with the QDs by fitting the PL lifetime data in Figure 2B to a model proposed by Tachiya et al. (details of the calculations are given in the Supporting Information).^45^ Using a global fitting procedure, we were able to obtain both the effective number of quenching clusters per QD, *m* (Figure 4A), and the effective rate of HT, *k*_HT_. As seen in Figures 2D and 4A, increasing the amount of MS added leads to more clusters interacting with the QD, consistent with the hypothesis that stripping capping ligands enhances surface accessibility and promotes interactions between the QD and **V**_**6**_ **O**_**7**_^**1–**^. Increased QD-cluster association is attributed to positively charged Cd sites formed following the removal of the oleate ligand with MS, enhancing electrostatic attractions between the nanocrystal surface and the monoanionic cluster.

**Figure 4:**
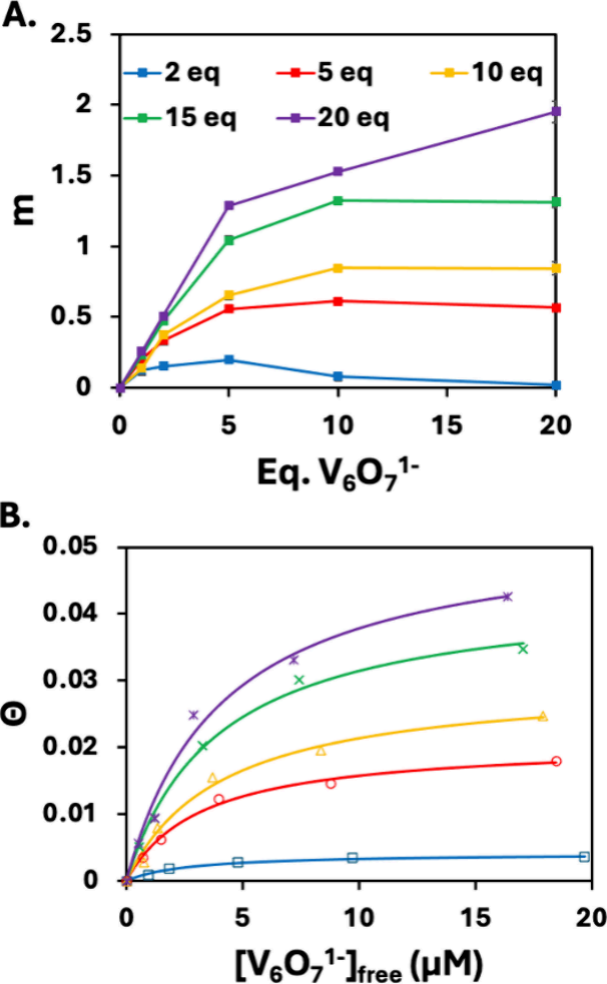
(A) Number of molecular acceptors interacting with the QDs estimated by fitting the time-resolved PL data at different concentrations of MS related to the equivalents of **V_6_O_7_^1–^** per QD. (B) Mean fractional surface coverage of **V_6_O_7_^1–^** on QDs (θ) as a function of the concentration of free **V_6_O_7_^1–^** across samples treated with varied equivalents of MS. Solid lines are fits derived using a Langmuir isotherm.

The observation that the increased removal of QD ligands by MS leads to a proportional increase in strongly associated QD-clusters suggests that a quantitative relationship can be established between the amount of added MS and accessibility of the nanocrystal surface. Previous work by Morris-Cohen et al. describes a simple Langmuir fitting model to provide a partial representation of the mean fractional surface coverage (θ) of an exchanged ligand on cadmium sulfide (CdS) QDs as a function of the exchanged ligand concentration.^32^ Here, we adapt a similar Langmuir isotherm model to quantify the adsorption of **V**_**6**_ **O**_**7**_^**1–**^ to the QD surface as a function of the added MS.

The relationship between the steady-state PL quenching values *I*/*I*_0_, the mean fractional cluster surface coverage (θ), and the total number of available binding sites per QD (*N*) is given by eq 1:^32^

1 $$\frac{I}{I_{0}}={\left(1-\theta \right)}^{N}$$

We define the parameter *N* to be the average number of oleate ligands per QD as quantified via ^1^H NMR spectroscopy with the rationale that, as MS strips native oleate ligands, these surface sites become accessible for cluster adsorption. Thus, *N* is directly related to the number of possible native ligands that can be removed from the surface upon treatment with MS. Analysis of the ^1^H NMR spectra of OA-capped CdSe QDs treated with increasing amounts of MS (Figure S7 and Table S1) provides an average of 85 native OA ligands. As such, a value of 85 was used for *N* (the total number of ligands at the surface of the nanocrystal prior to treatment with MS). Obtaining a value for *N* allows for calculation of the parameter θ, which can be expressed as a function of both added MS and **V**_**6**_ **O**_**7**_^**1–**^, which are shown in Figure 4B.

A Langmuir isotherm (eq 2) was used to fit the surface coverage data in Figure 4B:

2 $$\theta =\theta_{\max}\frac{K_{a}{\left[V_{6}{O_{7}}^{1-}\right]}_{\mathrm{free}}}{1+K_{a}{\left[V_{6}{O_{7}}^{1-}\right]}_{\mathrm{free}}}$$

where θ_max_ is the point of saturation of **V_6_O_7_^1–^** on the QD surface^32^ and [**V_6_O_7_^1–^**]_free_ is defined as follows: [**V_6_O_7_^1–^**]_free_ = [**V_6_O_7_^1–^**]_total_ – [**V_6_O_7_^1–^**]_bound_ = [**V_6_O_7_^1–^**]_total_ – [QD]*N*θ. As seen in Figure 4B, a Langmuir isotherm is an excellent model for describing the interaction between clusters and QDs in the presence of varying amounts of MS. In particular, the model allows for a quantitative relationship between the average QD surface coverage by **V_6_O_7_^1–^** and the amount of MS added. For example, at the lowest equivalents of MS added, the maximum fractional surface coverage of cluster (θ_max_) is 0.4%, while at 20 equiv of MS, θ_max_ increases to 5.3% (Figure 4B; see also Table S7). Our calculated θ_max_ at this condition is not 100%, which we attribute to the **V**_**6**_ **O**_**7**_^**1–**^ cluster being significantly larger than a single surface capping ligand. Indeed, the cluster radius is approximately one-ninth of the total QD surface area, and therefore even when a large fraction of the 85 “binding sites” become accessible via ligand removal, cluster coverage will not come close to reaching 100%.

A comparison of the results from the Langmuir isotherm fitting analysis to time-resolved PL data shows excellent agreement. As described above, the time-resolved PL experiments (Tables S2–S6) afford the quantification of molecular acceptors (**V**_**6**_ **O**_**7**_^**1–**^) that bind to the QD surface upon treatment with varied amounts of MS (Figure 4A), which can be directly compared to the fraction of available surface sites. For example, following treatment with 20 equiv of MS, we show that, after the addition of 20 equiv of **V**_**6**_ **O**_**7**_^**1–**^, approximately two clusters are bound to the QD surface as molecular acceptors. By comparison, the Langmuir analysis method provides a value of 4.25% for θ, which represents the percentage surface coverage by **V**_**6**_ **O**_**7**_^**1–**^ at this data point. Given that we have defined *N* as 85 possible surface sites, we use this value of θ to obtain an approximate 3.6 surface sites that are accessible to the cluster, a value that is quite close to the effective 2 clusters found to be bound to the nanocrystal surface, as calculated from the time-resolved PL data.

Overall, our results provide important context to understanding how QD surface accessibility and surface chemistry affect efficient HT. For example, for CdSe/CdS QDs, HT efficiencies of up to 99% are possible when controlling for shell thickness, ligand sterics, and the number of acceptor ligands.^28,46^ Rapid HT in under 1 ps from photoexcited CdS QDs to phenothiazine, facilitated by a phenyldithiocarbamate (PTC) linker, is possible due to delocalization of the hole outside the QD core and into the attached PTC ligand.^21^ Building on this understanding, our team previously showed that direct binding through the P=O moiety of TDPA ligands of CdSe QDs enables accelerated HT to oxygen-deficient POV-alkoxide clusters.^22^ This strategy highlighted how the design of the QD–ligand–acceptor interface could significantly influence the charge transfer processes. Both of these studies converge on a unifying principle: efficient charge transfer requires precise control of the nanomaterial surface to ensure close and effective contact between QD donors and molecular acceptor.

In conclusion, we have shown that achieving quantitative control over QD-based charge transfer processes necessitates a detailed accounting of the critical factor of surface accessibility. In our case, this control was achieved by adding precise equivalents of MS to OA-capped CdSe QDs, which can be used to strip the OA from the QD in a stochiometric manner. Further, we have established a quantitative relationship between the amount of MS added to the QDs and the fraction of the QD surface area that is made available for charge transfer reactions with freely diffusing molecular species. With increasing added MS comes increasing surface accessibility, which facilitates increased HT efficiency to **V_6_O_7_^1–^** through more facile cluster binding. Overall, these studies show that tuning the ligand density is a simple and straightforward parameter that can be used to adjust the outcome of photochemical reactions employing QDs as light-harvesting elements, or as direct photocatalysts.

## Supplementary Material



## Notes

QD synthesis and characterization, details of experimental procedures and data interpretation, and ^1^H NMR and PL spectra (PDF)
