# Supplementary material for: Modulating Hole Transfer from CdSe Quantum Dots by Manipulating the Surface Ligand Density
Source: Nano Lett. 2025 May 23;25(22):8993–8. doi: 10.1021/acs.nanolett.5c01323 (PMC12142658; doi:10.1021/acs.nanolett.5c01323)
Supplement: Supplementary file 1 [file nl5c01323_si_001.pdf]

Modulating hole transfer from CdSe quantum dots by manipulating surface ligand density

Chari Y.M. Peter[a], Chayan Carmenate Rodríguez[a], Hannah N. Gorski[a], Elizabeth O. Phinney[a], Todd D. Krauss[a,b]*, Ellen M. Matson[a]*

[a]*Department of Chemistry, University of Rochester, Rochester NY, 14627 USA.*

[b]*Institute of Optics, University of Rochester, Rochester NY, 14627 USA.*

Corresponding Author Contact Information:

Todd D. Krauss: todd.krauss@rochester.edu

Ellen M. Matson: matson@chem.rochester.edu

Table of Contents

Experimental procedures and calculations

Synthesis of OA-CdSe QDs S3

Synthesis of [^{n}Bu$_4$N][$V_6O_7(OCH_3)_{12}$]: $V_6O_7^{1-}$.......... S3

Time Correlated Single Photon Counting S4

Transient Absorption Spectroscopy S4

OA-CdSe + MS Quantitative ^{1}H NMR S5

Hole Transfer Efficiency Calculations S5

Energy Transfer Calculations S9

PL Decay Dynamics Analysis S21

Molecular Acceptors Analysis S21

Fraction Accessible Analysis S28

Supporting Figures

Figure S1: TEM of OA CdSe – 0eq + 20eq MS S6

Figure S2. Steady-State PL $V_6O_7^{1-}$ + OA CdSe QDs S7

Figure S3. Steady-State PL $V_6O_7^{1-}$ + OA CdSe QDs +10eq Oleic acid.......... S7

Figure S4. Redox Potentials of OA-CdSe Relative to $V_6O_7^{1-}$ S8

Figure S5. Absorption Spectrum of Cluster & Emission of QDs S9
Figure S6. Transient Absorption Dynamics S10
Figure S7: ^{1}H NMR Spectra of QDs + 0-20eq MS S11
Figure S8: ^{1}H NMR Spectra of QDs + 0 and 100eq MS.. S12
Figure S9: Peak Analysis of ^{1}H NMR Spectra of QDs + MS S13
Figure S10. Steady-State PL & Time-Resolved PL Kinetics of QDs + 0eq MS S15
Figure S11. Steady-State PL & Time-Resolved PL Kinetics of QDs + 2eq MS S16
Figure S12. Steady-State PL & Time-Resolved PL Kinetics of QDs + 5eq MS S17
Figure S13. Steady-State PL & Time-Resolved PL Kinetics of QDs + 10eq MS S18
Figure S14. Steady-State PL & Time-Resolved PL Kinetics of QDs + 15eq MS S19
Figure S15. Quenching Efficiencies of QDs + 20eq MS S20

Supporting Tables

Table S1: Quantified Ligand Coverage on QD as Calculated by ^{1}H NMR S14
Table S2. Summary of TCSPC Fitting Parameters for QDs + 2eq MS S22
Table S3. Summary of TCSPC Fitting Parameters for QDs + 5eq MS S23
Table S4. Summary of TCSPC Fitting Parameters for QDs + 10eq MS S24
Table S5. Summary of TCSPC Fitting Parameters for QDs + 15eq MS S25
Table S6. Summary of TCSPC Fitting Parameters for QDs + 20eq MS S26
Table S7: Langmuir Parameters Summary S27

Experimental Methods:

Chemicals were purchased and used without further purification except where noted. Cadmium (II) Cadmium Oxide (CdO, 99.99%), Selenium (Se, 100 mesh, 99.99%), 1-Octadecene (ODE, 90%), Oleic Acid (OA, 90%), Trimethyl oxonium tetrafluoroborate (95%) and Tetrabutylammonium Borohydride ($TBABH_4$, 98%), were purchased from Sigma-Aldrich. Tetrabutylammonium Hexafluorophosphate (TBA PF6, 98%) was purchased from Sigma-Aldrich, recrystallized thrice using hot methanol, and stored under dynamic vacuum for a minimum of two days prior to use.

OA-CdSe QDs Synthesis:

To a dry, three- neck round bottom flask, 3ml of OA, 10ml of Ode and 0.514g of CdO (0.514g, 4.00mmol) were added. The flask was placed under an N_2 atmosphere and heat to 260°C while stirring at 1100 rpm under reflux. While the solution was heating, the Se precursor was prepared by sonication 0.68g of Se in 10 ml ODE until a uniform suspension was formed. Once the Cd precursor became clear and approached -260°C, **1 mL Se** precursor was rapidly injected. The QDs were allowed to grow for 20 seconds before thermal quenching with a water bath. In order to remove the unused precursor, the solution was washed with ethanol in a 6:1 (v/v) ratio to QDs followed by centrifugation at 5000 rpm for 15 minutes. The clear supernatant was then discarded, and the pellet was redispersed in minimal hexanes. We repeated the washing once more and stored the final QD solution in 15mL Hexanes.

Characterization of Nanocrystals: Transmission electron microscopy (TEM) measurements were performed using FEI TECNAI F-20 field emission microscope (accelerating voltage of 200 kV) on -400 mesh lacey carbon grids. 1ml of stock OA-CdSe QDs were washed with ethanol six times and diluted in hexane. The diluted QDs were then drop-casted onto the carbon grid and allowed to dry overnight before imaging. This was repeated for 20eqMS + QDs. ImageJ was used to determine the size distribution of the imaged QDs where the data was plotted using MATLAB.

Synthesis of [$^nBu_4N$][$V_6O_7(OCH_3)_{12}$]:

$V_6O_7^{-1}$ was prepared according to previous literature.[1]

Time-Correlated Single Photon Counting

Ensemble TCSPC measurements were acquired using a home-built optical setup featuring an inverted microscope (Nikon, TE-200U) with a 10x air objective (Nikon, NA 0.3) and a 488 nm dichroic mirror (Semrock, Inc.). Samples, which had been transferred to quartz cuvettes with 1 mm thick windows, were photoexcited by a defocused laser beam provided by a pulsed laser diode (PicoQuant, PDL-800D) emitting 485 nm light with a repetition frequency of 5 MHz. The laser power was adjusted to ensure the photon flux was no higher than 10^{12} photons per cm^2 per pulse. Photoluminescence (PL) emission was collected by a single photon avalanche diode detector (Micro Photon Devices, PDM Series, 50 µm active sensing area diameter) with a fast-timing resolution lower than 100 ps. All components of the TCSPC setup were incorporated in the PicoHarp 300 module (PicoQuant). The instrument response function was collected from residual scattering of the light from the pulsed laser diode. To collect lifetime decay curves, 488 nm and 532 nm longpass filters were placed in the emission pathway to block scattered excitation light. Decay curves corresponding to OA-CdSe QDs + n eq MS without the quencher were first fit to a triple exponential decay to establish the dynamics before PL quenching was initiated. Subsequent decay curves corresponding to OA-CdSe QDs + n eq MS with the quencher were fit according to the lifetime parameters calculated for the pre-quenching sample and an additional, floating parameter used to examine the quenching process.

Transient Absorption

TA measurements were performed using an ultrafast laser system in a pump-probe configuration. Femtosecond pulses (277 fs) from a 1035 nm diode-pumped fiber laser (Coherent, Monaco IR) at 500 kHz were introduced into an optical parametric amplifier (Coherent, Opera-NOPA). The resulting signal pulses (≈ 50 fs, 500 kHz) were tuned to the desired wavelength (535 nm) and used as a pump. Probe beam was generated using a YAG crystal pumped by the fundamental 1035 nm from Monaco IR. The reference beam was split from the probe before the sample and used to correct the probe for pulse-to-pulse fluctuations. A motorized delay stage was used to control the optical time delay between the pump and probe pulses. Samples were placed in a 2 mm path length quartz cuvette and stirred to avoid laser-induced signal artifacts. The optical density of CdSe QDs was 0.1 for at the pump wavelength of 535 nm. The average number of excitons absorbed per QD, $\langle N_0 \rangle$, was kept below one for all measurements.

OA-CdSe + Meerwein's Salt Quantitative ^{1}H NMR Spectroscopy

All NMR samples were prepared outside of a glovebox. NMR samples were prepared by first drying aliquots from a QD stock that would yield **100 μM** samples under an air stream. A ferrocene internal standard solution was prepared by dissolving **5 mg of ferrocene** in 500 μL of toluene-d_8. A 4 mM stock solution of Meerwein's salt in DCM was prepared inside a nitrogen filled glove box. To the dried QDs, an appropriate volume of DCM and the Meerwein's salt stock solution was added such that the total volume reaches 600 μL for all samples (0, 2, 5, 10, 15, 20, 40 equivalents Meerwein's salt). All samples were left to sit for 1 hour in the DCM suspensions, then dried under an air stream and resuspended in 590 μL toluene-d_8 and 10 μL of the ferrocene internal standard solution was added to each NMR tube. All ^{1}H NMR spectra were collected on a Bruker 400 MHz spectrometer.

Following spectra collection, quantification of bound oleic acid and free methyl oleate was obtained via peak fitting in MestReNova. Figure S2 (A-F) displays the fitting peaks in blue, the total fit in pink, and the fit residual in red. To obtain quantitative values, the respective areas of bound or free ligand peaks were summed and converted to number of ligand/QD against the ferrocene internal standard. These values are reported in Table S1.

Hole transfer efficiencies calculations

Hole transfer efficiency derived from PL lifetimes can be calculated by taking the ratio of the integrated area under the time-resolved PL decay curves with and without the cluster according to:[2]

$$E_{HT}^{lifetimes} = 1 - \frac{\int PL(QDs + MS + V_6O_7^{1-})}{\int PL(QDs + MS)} \qquad (1)$$

And the hole transfer efficiency based in steady-state (quenching) is defined as:

$$E_{HT}^{quenching} = 1 - \frac{I}{I_0} \qquad (2)$$

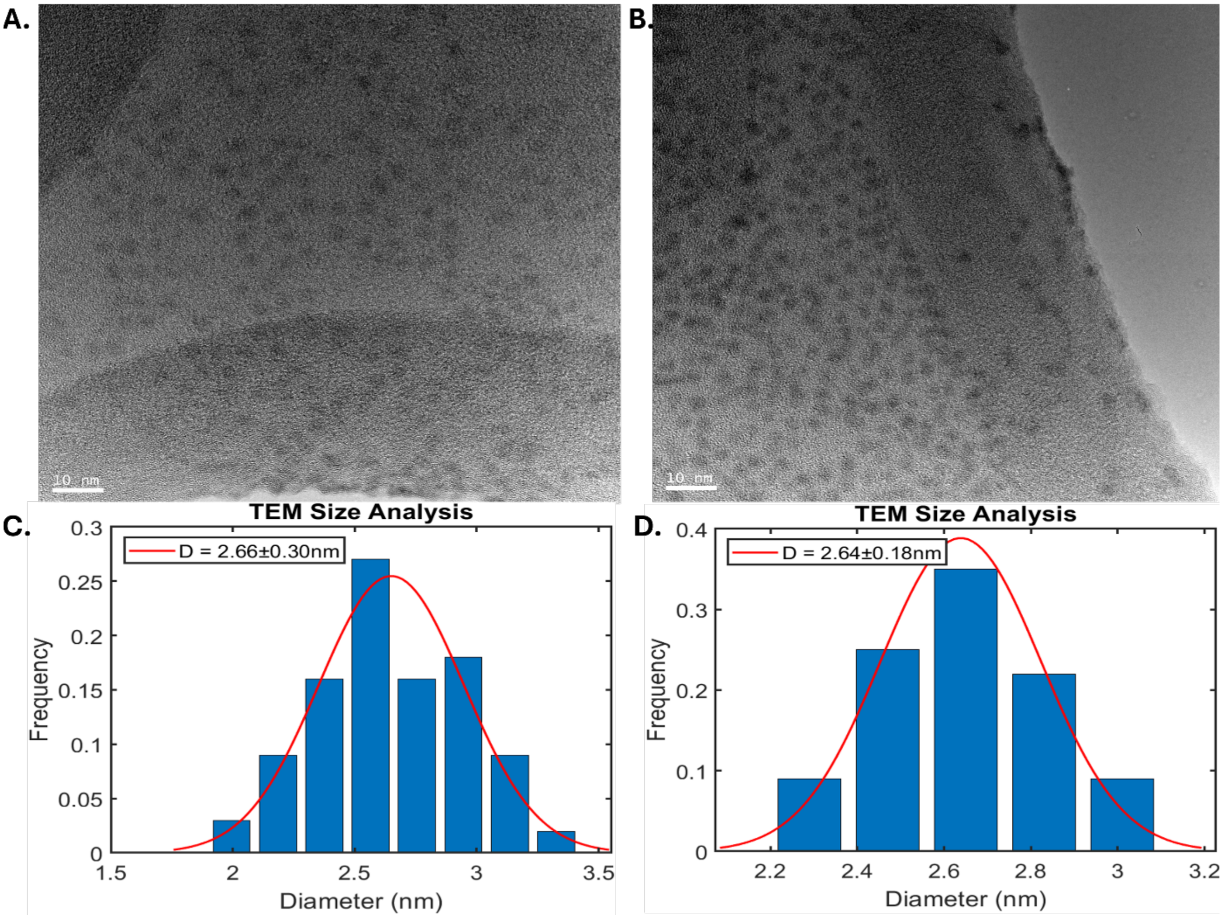

Figure S1. TEM of (A) OA-CdSe QDs, (B) OA-CdSe QDs following addition of 20eq MS. (C) Size distribution of the imaged OA CdSe QDs (D) Size distribution of the imaged OA CdSe QDs following addition of 20eq MS.

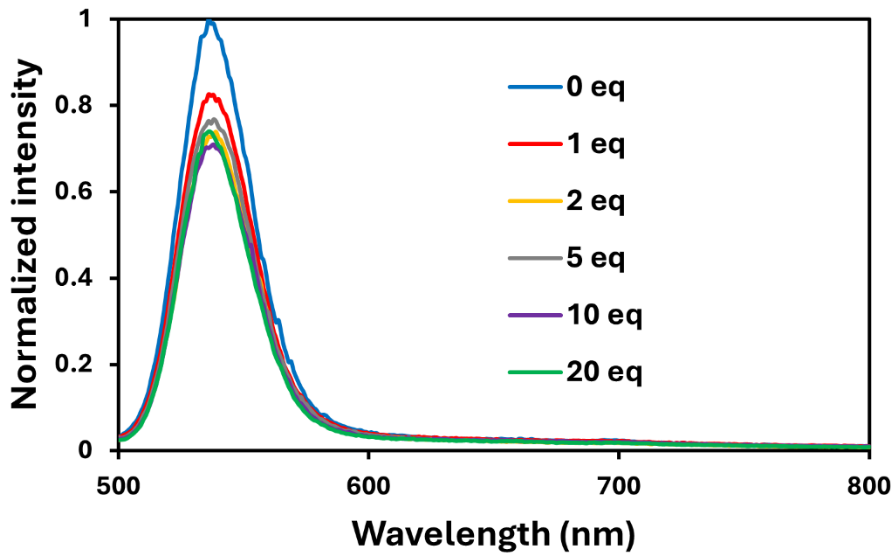

Figure S2. Normalized PL intensity of $V_6O_7^{1-}$ + OA CdSe.

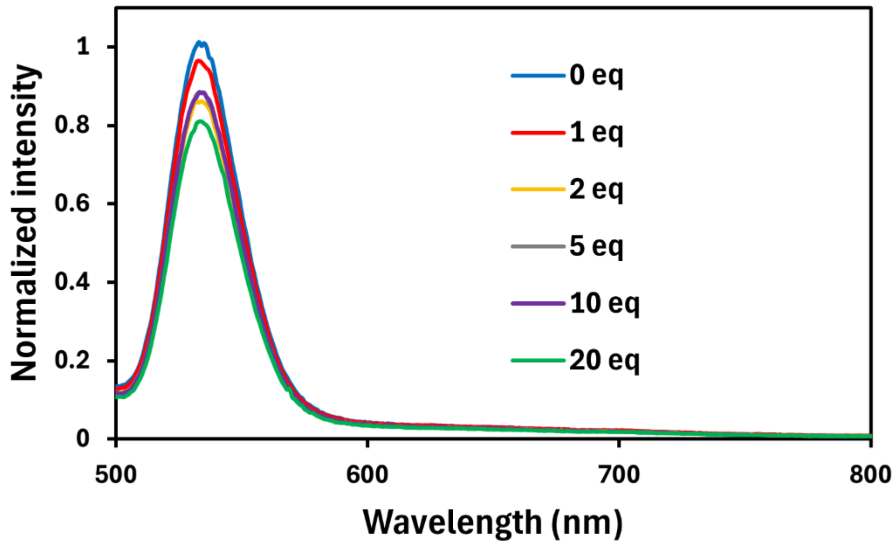

Figure S3. Control experiment: Normalized PL intensity of $V_6O_7^{1-}$ + 10eq OA + OA CdSe. The addition of 10eq of free oleic acid to $V_6O_7^{1-}$ + OA CdSe reduced quenching efficiency in comparison to the absence of free ligand. This is attributed to the enhanced surface passivation of the native NC by OA.

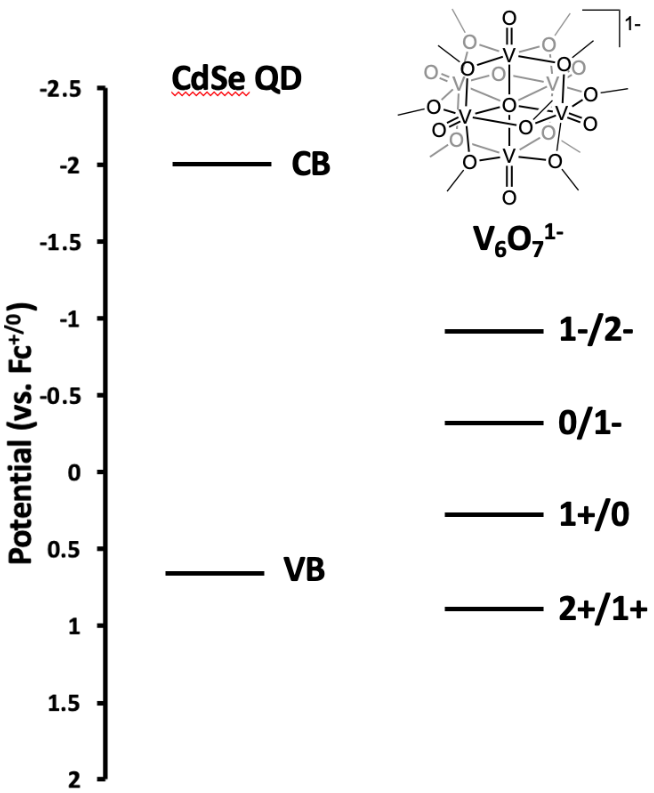

Figure S4: Estimated valence and conduction band potentials for CdSe QDs as compared to reported redox potentials for the $V_6O_7^{1-}$ cluster in DCM.[4]

Energy transfer calculations[3,5]

To evaluate the occurrence of energy transfer as an additional pathway of fluorescence quenching of the QDs, we need to analyze the overlap between the emission of the donor (QDs + 20 eq MS) and the absorption of the acceptor ($V_6O_7^{1-}$ clusters).

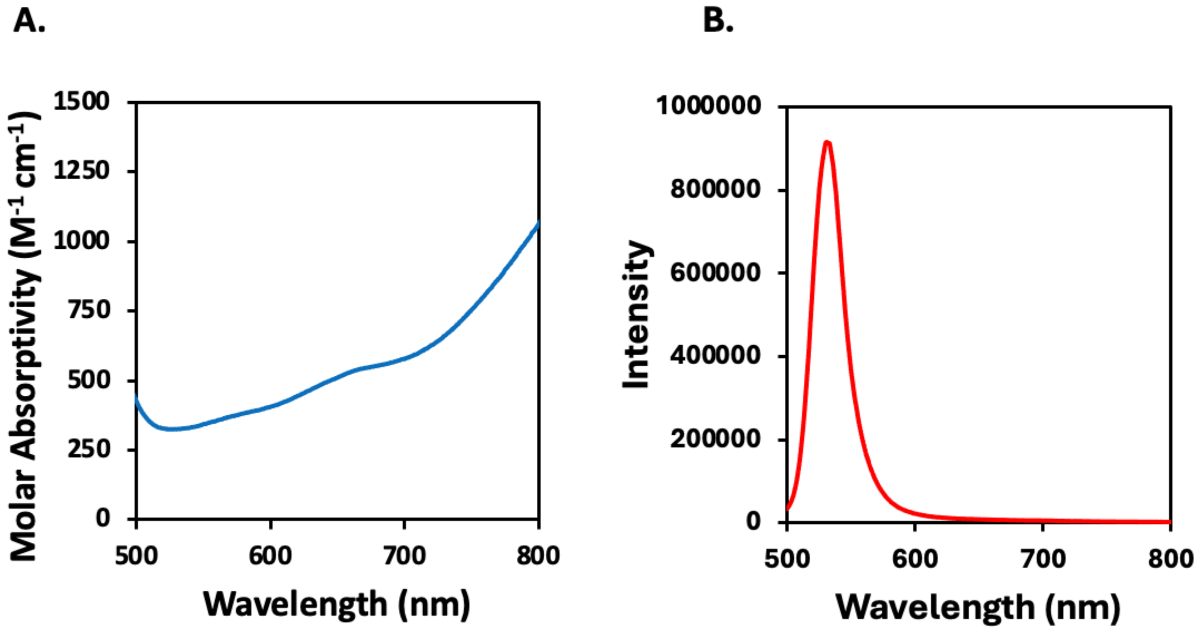

Figure S5. (A) Absorption spectrum of the acceptor ($V_6O_7^{1-}$ clusters). (B) Emission spectrum of the donor (QDs + 20 eq MS).

Spectral overlap integral as a function of wavelength is defined as:

$$J(\lambda) = \int \varepsilon_A(\lambda) F_D(\lambda)\, \lambda^4 d\lambda \qquad (1)$$

In equation (1), units of J are $M^{-1}\ cm^{-1}\ nm^4$, ε_A is the extinction coefficient spectrum of the acceptor and F_D is the wavelength dependent donor emission spectrum normalized to an area of 1. From this we obtain an overlap of 3.05 x 10^{13} $M^{-1}\ cm^{-1}\ nm^4$.

Consider the Förster radius R_0 in nm as the donor-acceptor distance at which the energy transfer efficiency is 50%:

$$R_0 = 0.0211 \left(\frac{\kappa^2 \Theta_D J(\lambda)}{n^4} \right)^{\frac{1}{6}} \qquad (2)$$

Where κ is the dipole orientation factor (2/3 for these quasi-spherical structures), Θ_D is the fluorescence QY for the QDs + 20 eq MS without cluster present (5.17%, measured previously)

and n is the refractive index of DCM (1.4244). Using the result obtained in (1), we determined R_0 = 1.57 nm.

Finally, the energy transfer efficiency E can be calculated as:

$$E = \frac{1}{1 + \left(\frac{R}{R_0}\right)^6} \qquad (3)$$

Where R is the donor-acceptor distance in nm. In this case we assumed R as the smallest center-to-center distance possible of 2.5 nm. Thus, we obtained an energy transfer efficiency of 5.8%. This value, compared with the 40% quenching observed with one equivalent of clusters added, makes the hole transfer the dominant pathway of fluorescence quenching in the system.

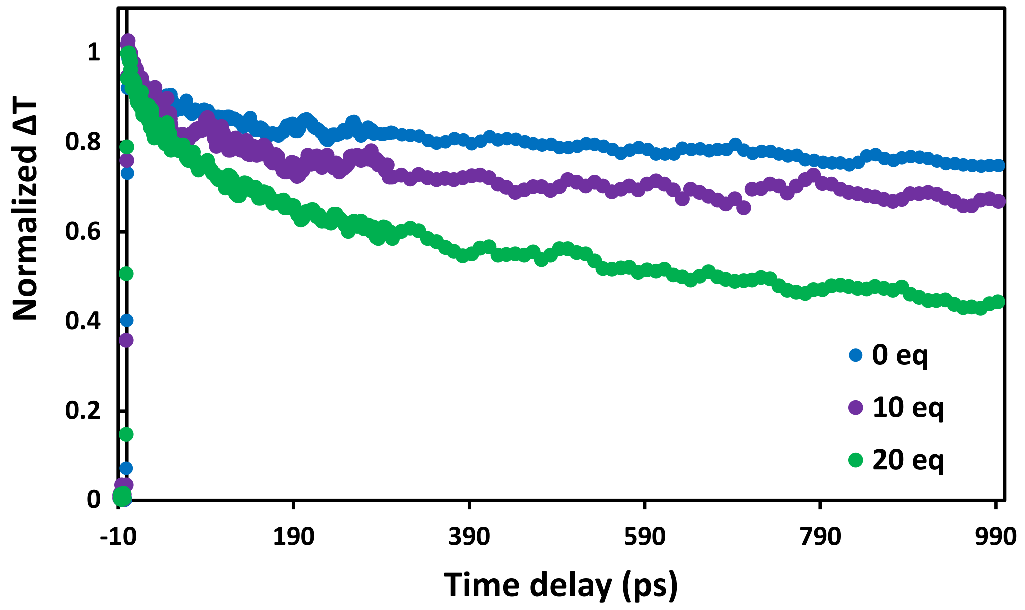

Figure S6. Normalized change in transmission (ΔT) of the $1S_e$–$1S_{h3/2}$ transition of stripped OA-CdSe QDs (20 eq Meerwein's salt) without $V_6O_7^{1-}$ present (blue) and with 10 eq (purple) and 20 eq of $V_6O_7^{1-}$ (green) as a function of probe delay up to 1000 ps.

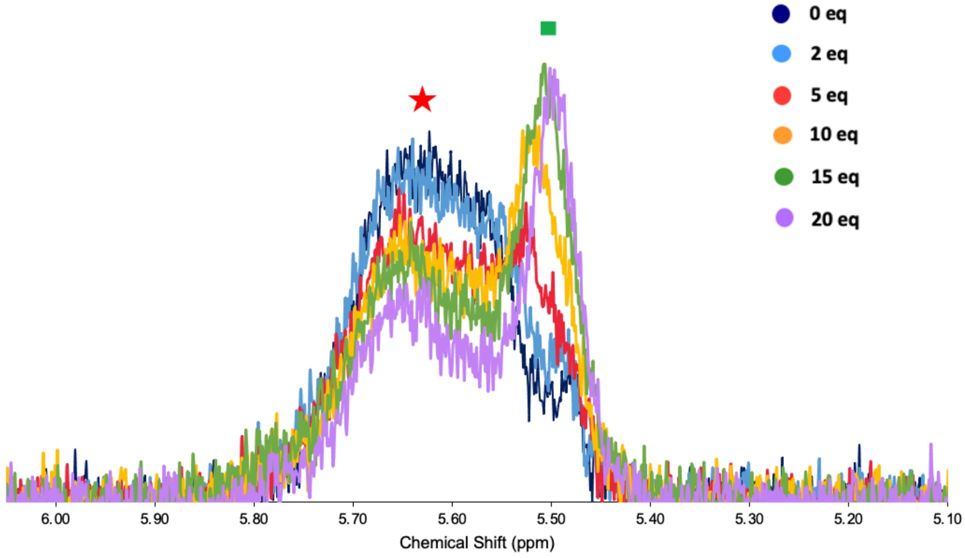

Figure S7. ^{1}H NMR spectra of OA-CdSe QDs with 0-20eq MS in toluene-d_8. Shown is the alkene region of the full spectrum which displays both bound OA (red star) and free methyl oleate (green square). Analysis of the alkene signal from OA allows for the ability to distinguish between ligands bound to the nanocrystal surface and those that are free based on their relative broadening and chemical shift in deuterated aromatic solvents. This is predominantly due to the differences of chemical environment for bound and free ligand species. OA bound to the QD surface tumbles more slowly through solution as compared to free methyl oleate, which tumbles at a faster rate and therefore exhibits signal sharpening in addition to an aromatic solvent-induced shift in toluene-d_8.[6,7,8]

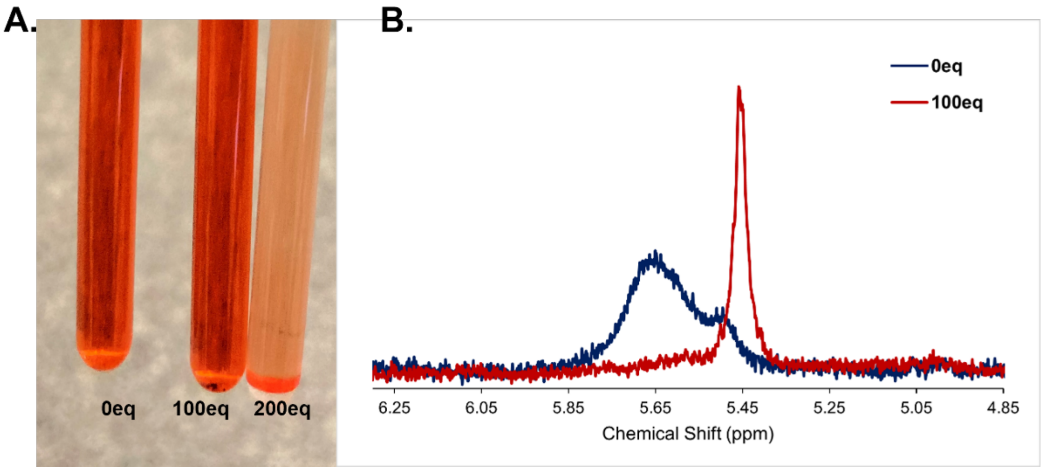

Figure S8. A) Image of CdSe QDs depicting change in solubility of nanoparticles with higher equivalents of MS **B)** ^{1}H NMR spectra of OA-CdSe QDs with 0 and 100eq MS in toluene-d_8. Shown is the alkene region of the full spectrum which displays both bound OA (blue) and free methyl oleate (red). At 100eq all of the bound OA is completely stripped off the QD and appears to lose colloidal stability.

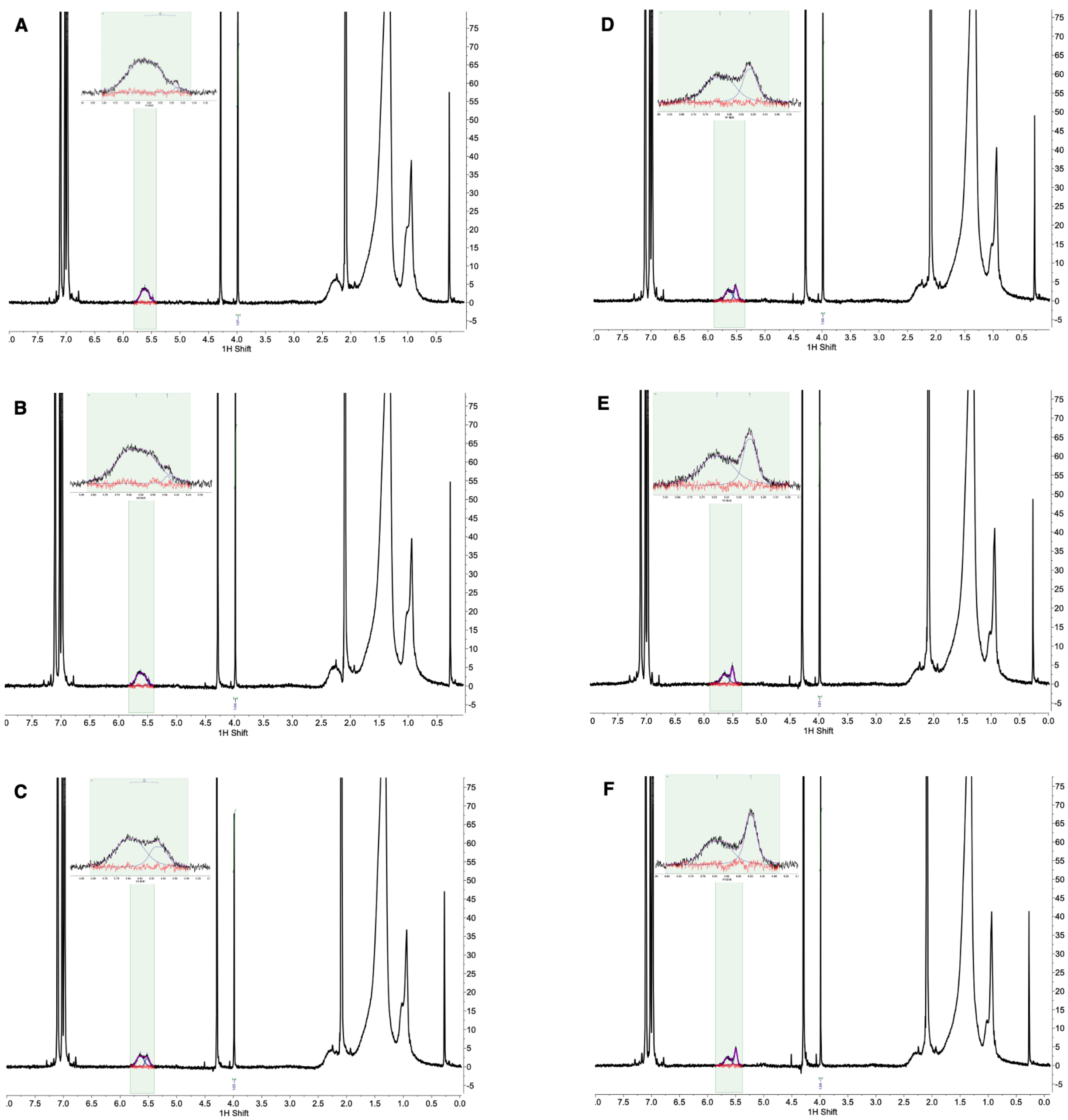

Figure S9. ^{1}H NMR spectra of OA-CdSe QDs in toluene-d_8 upon treatment with A) 0eq MS, B) 2eq MS, C) 5eq MS, D) 10eq MS, E) 15eq MS, and F) 20eq MS. The peak fitting region is highlighted in green. Within the peak fitting region, fitting peaks are in blue, the total fit is pink, and the fit residual fit is in red.

Table S1. Quantified bound OA per QD, free methyl oleate per QD, and total ligand per QD as calculated via ^{1}H NMR peak fitting and integration in MestReNova against a ferrocene internal standard.

	**Bound OA/QD**	**Free Methyl Oleate/QD**	**Total Ligand/QD**
**QD Only**	81	2	83
**2eq MS**	87	4	91
**5eq MS**	61	26	87
**10eq MS**	50	35	85
**15eq MS**	56	34	91
**20eq MS**	38	38	76
		**Average Ligand/QD**	85 ± 6

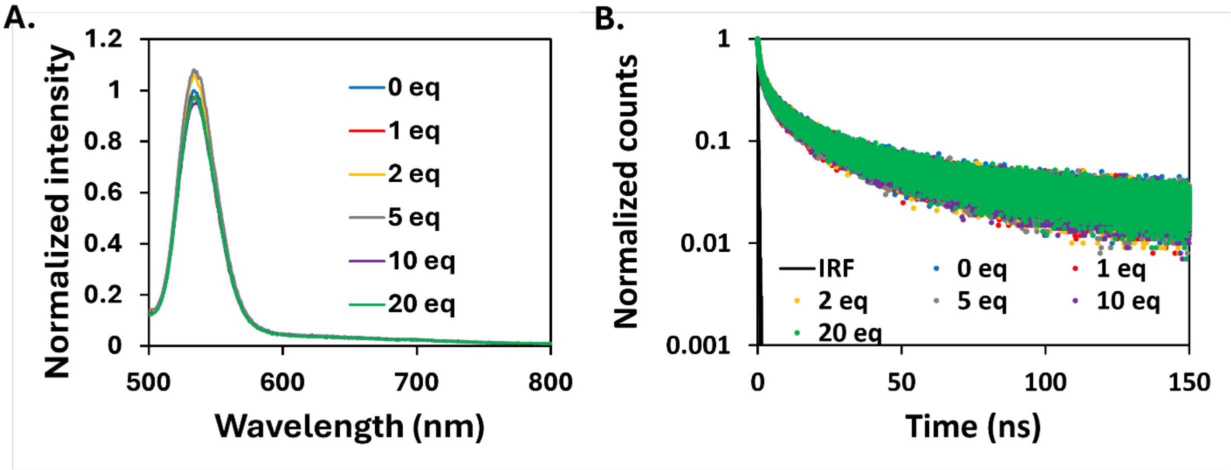

Figure S10. (A) Normalized PL intensity and (B) normalized PL kinetics of OA-CdSe QDs with an increasing number of $V_6O_7^{1-}$ equivalents per QD added.

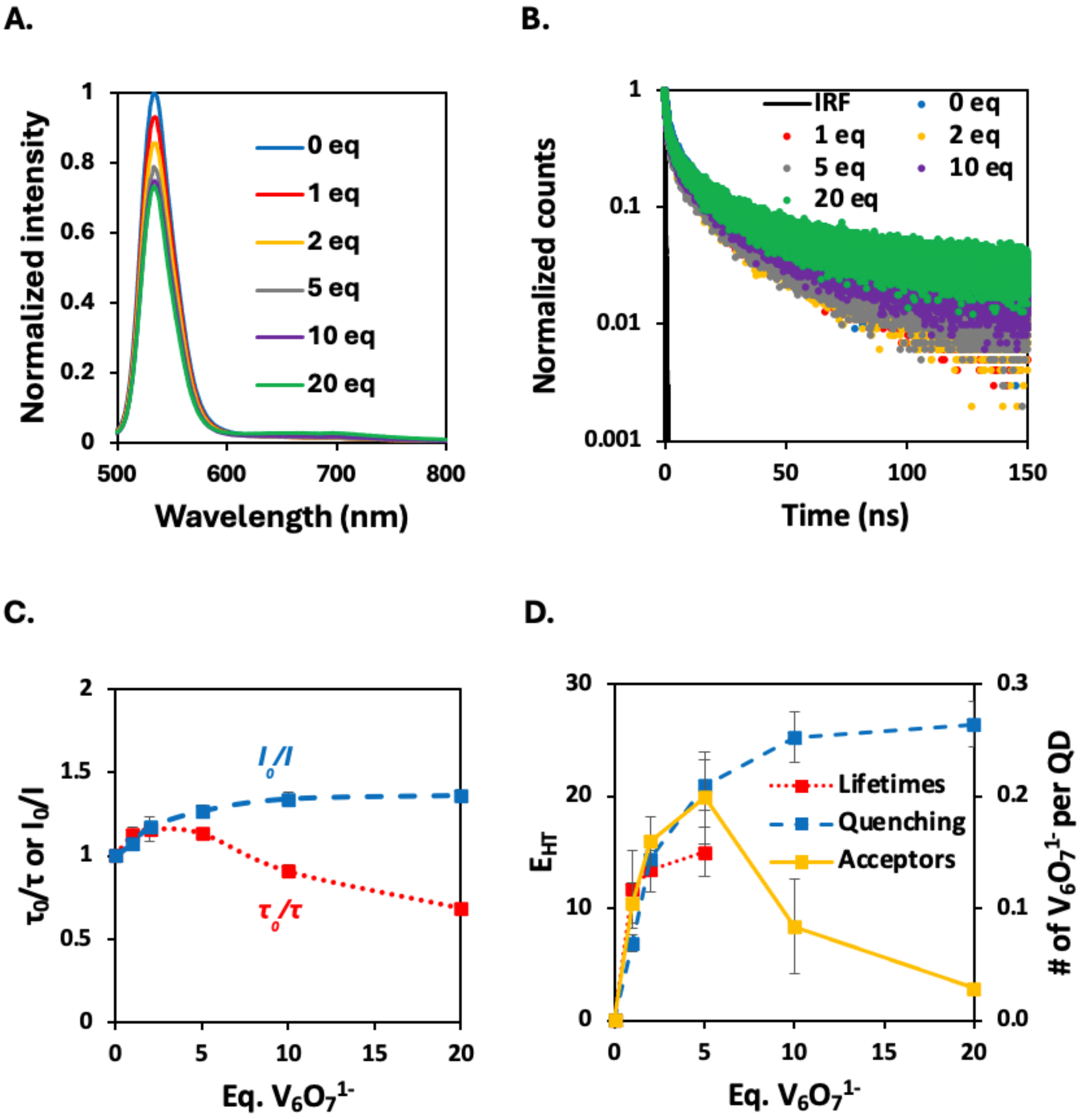

Figure S11. (A) Normalized PL intensity and (B) normalized PL kinetics of OA-CdSe QDs + 2 eq of Meerwein's salt with an increasing number of $V_6O_7^{1-}$ equivalents. (C) Steady-state (blue) and time-resolved (red) PL quenching. Both the red dotted line and blue dashed line are a guide to the eye. τ_0 is the amplitude-weighted average PL lifetime of just the QDs + MS, and τ is the lifetime with added $V_6O_7^{1-}$. (D) PL quenching efficiency based on steady-state $E_{HT}^{(quenching)}$ (blue dashed line) and time-resolved $E_{HT}^{(lifetimes)}$ (red dotted line) measurements and calculated average numbers of acceptors per QD (orange solid line), which are related to the number of equivalents of $V_6O_7^{1-}$.

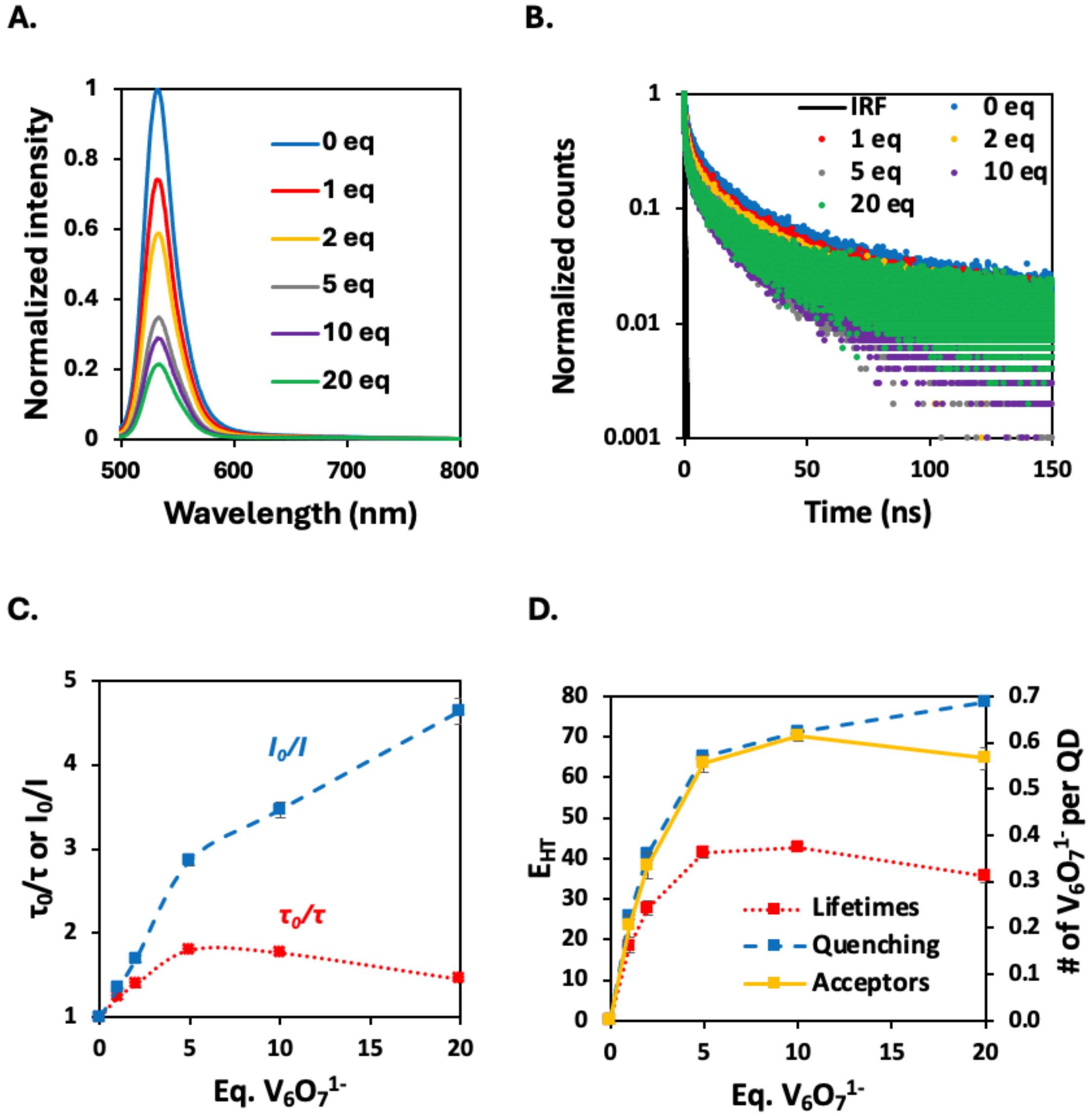

Figure S12. (A) Normalized PL intensity and (B) normalized PL kinetics of OA-CdSe QDs + 5 eq of Meerwein's salt with an increasing number of $V_6O_7^{1-}$ equivalents. (C) Steady-state (blue) and time-resolved (red) PL quenching. Both the red dotted line and blue dashed line are a guide to the eye. τ_0 is the amplitude-weighted average PL lifetime of just the QDs + MS, and τ is the lifetime with added $V_6O_7^{1-}$. (D) PL quenching efficiency based on steady-state $E_{HT}^{(quenching)}$ (blue dashed line) and time-resolved $E_{HT}^{(lifetimes)}$ (red dotted line) measurements and calculated average numbers of acceptors per QD (orange solid line), which are related to the number of equivalents of $V_6O_7^{1-}$.

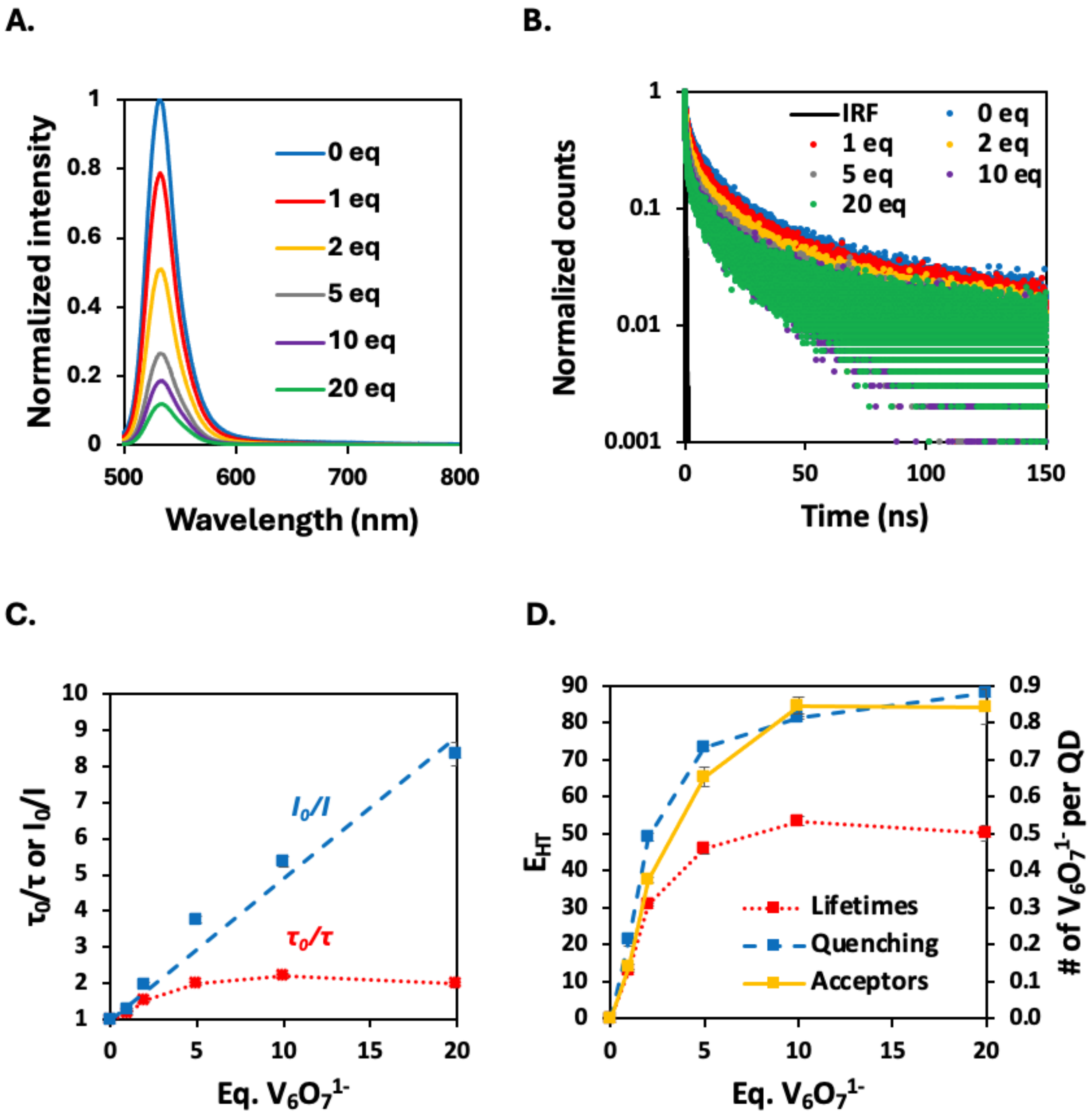

Figure S13. (A) Normalized PL intensity and (B) normalized PL kinetics of OA-CdSe QDs + 10 eq of Meerwein's salt with an increasing number of $V_6O_7^{1-}$ equivalents. (C) Steady-state (blue) and time-resolved (red) PL quenching. The red dotted line is a guide to the eye, while the blue dashed line is a linear fit. τ_0 is the amplitude-weighted average PL lifetime of just the QDs + MS, and τ is the lifetime with added $V_6O_7^{1-}$. (D) PL quenching efficiency based on steady-state $E_{HT}^{(quenching)}$ (blue dashed line) and time-resolved $E_{HT}^{(lifetimes)}$ (red dotted line) measurements and calculated average numbers of acceptors per QD (orange solid line), which are related to the number of equivalents of $V_6O_7^{1-}$.

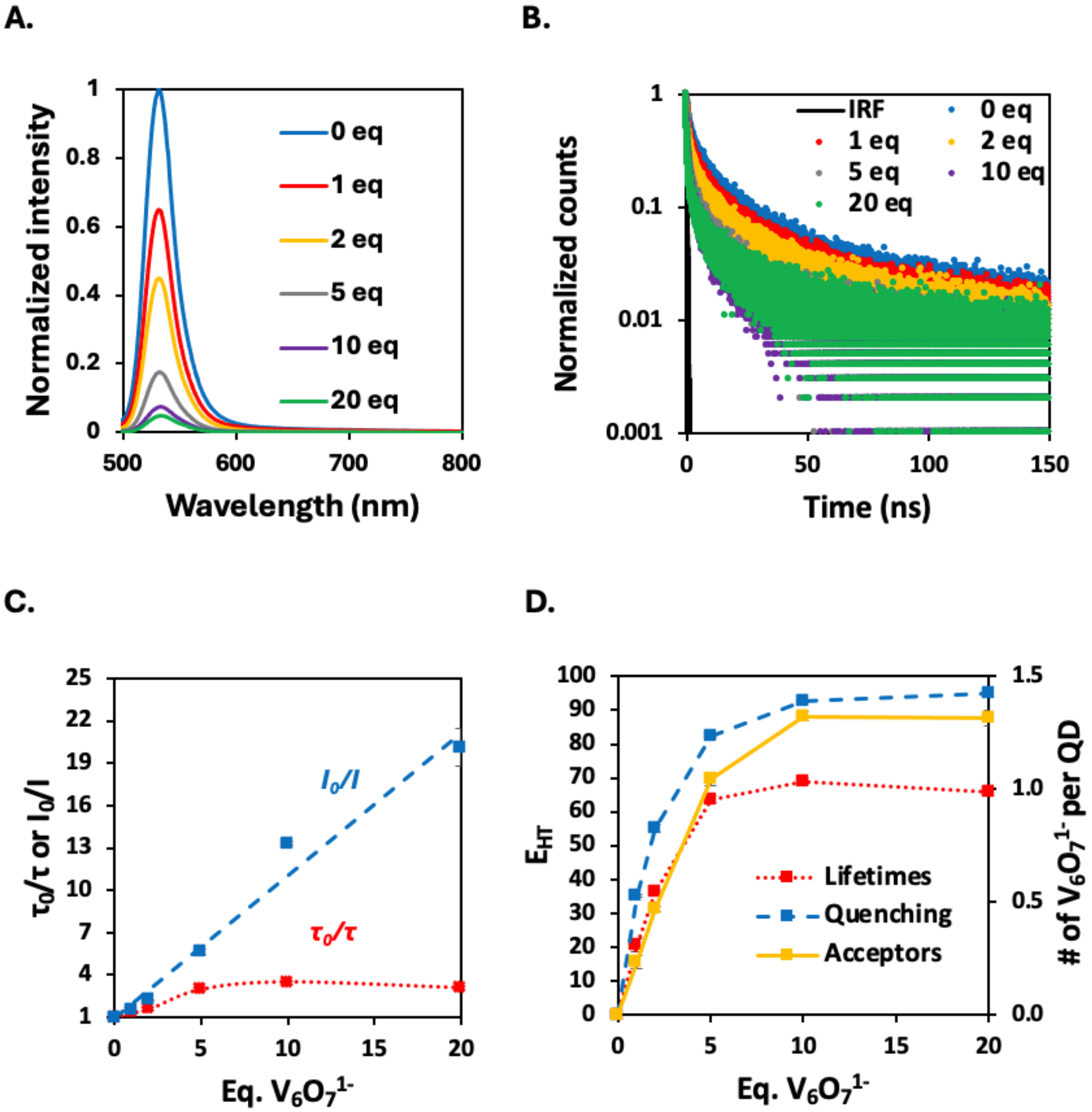

Figure S14. (A) Normalized PL intensity and (B) normalized PL kinetics of OA-CdSe QDs + 15 eq of Meerwein's salt with an increasing number of $V_6O_7^{1-}$ equivalents. (C) Steady-state (blue) and time-resolved (red) PL quenching. The red dotted line is a guide to the eye, while the blue dashed line is a linear fit. τ_0 is the amplitude-weighted average PL lifetime of just the QDs + MS, and τ is the lifetime with added $V_6O_7^{1-}$. (D) PL quenching efficiency based on steady-state $E_{HT}^{(quenching)}$ (blue dashed line) and time-resolved $E_{HT}^{(lifetimes)}$ (red dotted line) measurements and calculated average numbers of acceptors per QD (orange solid line), which are related to the number of equivalents of $V_6O_7^{1-}$.

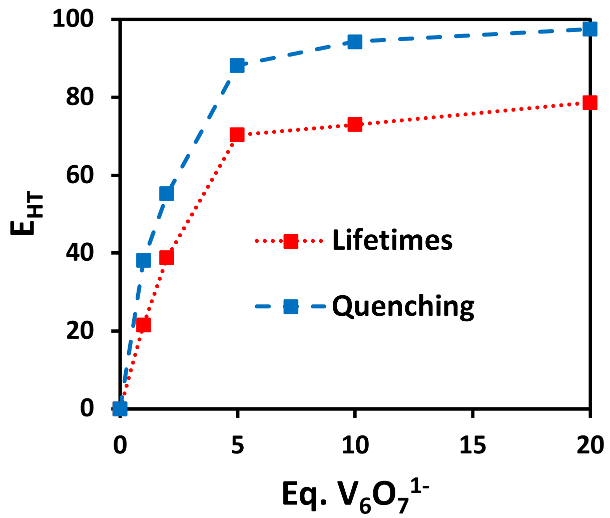

Figure S15. PL quenching efficiency of OA-CdSe QDs + 20 eq of Meerwein's salt with an increasing number of $V_6O_7^{1-}$ equivalents based on steady-state $E_{HT}^{(quenching)}$ (blue dashed line) and time-resolved $E_{HT}^{(lifetimes)}$ (red dotted line) measurements.

PL decay dynamics analysis

Intrinsic QD dynamics were obtained by fitting the kinetics of QDs + n eq MS (*n = 2-20*) without clusters to yield the amplitudes A_i and time constants τ_{QDi} ($\tau_{QDi} = 1/k_{QDi}$). The kinetics of the QDs + n eq MS with various $V_6O_7^{1-}$ cluster concentrations was globally fit, holding the amplitudes A_i and rate constants k_{QDi} of the intrinsic electron relaxation constant. To account for the HT process, amplitudes B_j and number of acceptors *m* were allowed to vary, while the rate constant k_{HTj} was held constant, as it denotes the rate for a 1:1 cluster to QD + n eq MS system. The intrinsic QD + n eq MS dynamics and the HT process are treated independently, and thus $\sum_i A_i = 1$ and $\sum_j B_j = 1$.

Number of molecular acceptors estimation

We estimate the number of molecular acceptors ($V_6O_7^{1-}$) interacting with the QDs by fitting the PL lifetime data to a model proposed by Tachiya et al.[3]. This model assumes a Poissonian distribution of quencher per chromophore given by:

$$f_n = \frac{m^n}{n!} e^{-m} \qquad (1)$$

where f_n is the probability of a given QD being quenched by *n* $V_6O_7^{1-}$ molecules and $m=\langle n \rangle$[2–4]. The total rate of hole transfer is the product of the effective number of POV-alkoxides per QD (*n*) and the hole transfer rate for one cluster per QD:

$$k_{HT-total} = nk_{HT} \qquad (2)$$

Therefore, the PL decay of excited QDs as a function of time is represented by the equation:

$$I(t) = I_0(\sum_i A_i e^{-k_{QD_i} t})(\sum_{n=0}^{\infty} f_n e^{-nk_{HT} t}) \qquad (3)$$

I_0 is a normalization constant corresponding to the initial concentration of excited QDs and A_i is the amplitude of the ith component of the QD PL decay without a cluster. Mathematically can be demonstrated that the sum corresponding to the hole transfer process (right sum in equation 3) can be simplified to:[3]

$$I(t) = I_0(\sum_i A_i e^{-k_{QD_i} t})[e^{(-m+me^{-k_{HT} t})}] \qquad (4)$$

Table S2. Fitting parameters for TCSPC lifetime measurements of OA-CdSe QDs + 2 eq MS with $V_6O_7^{1-}$ clusters.

**Intrinsic Relaxation Kinetics**				**Hole Transfer Kinetics**
**τ_{QD1}**<br>***A_1***	**τ_{QD2}**<br>***A_2***	**τ_{QD3}**<br>***A_3***	**$\langle\tau_{QD}\rangle$***	**τ_{1HT}**<br>***B_1***
0.97 ± 0.03 ns<br>*55.11 ± 1.99 %*	9.61 ± 0.17 ns<br>*35.10 ± 1.51 %*	69.94 ± 0.85 ns<br>*9.79 ± 0.48 %*	10.75 ± 0.39 ns	0.24 ± 0.03 ns<br>*100 %*

**Hole Transfer Kinetics with Various $V_6O_7^{1-}$ Equivalents**		
**# Equiv. $V_6O_7^{1-}$**	**# acceptors**	**τ_{nHT}#**
**1**	0.10 ± 0.02	2.32 ± 0.69 ns
**2**	0.16 ± 0.02	1.51 ± 0.39 ns
**5**	0.20 ± 0.04	1.24 ± 0.40 ns
**10**	0.08 ± 0.04	3.35 ± 2.05 ns
**20**	0.03 ± 0.00	8.33 ± 2.06 ns

*Amplitude-weighted average lifetime $\langle\tau_{QD}\rangle = \frac{\sum_i A_i \tau_{QDi}}{\sum_i A_i}$. #Hole transfer lifetime ($\frac{1}{k_{nj}} = \frac{n}{k_{1HT}}$). All errors are estimated by the standard deviation of multiple measurements.

Table S3. Fitting parameters for TCSPC lifetime measurements of OA-CdSe QDs + 5 eq MS with $V_6O_7^{1-}$ clusters.

Intrinsic Relaxation Kinetics				Hole Transfer Kinetics
τ_{QD1} ***A_1***	τ_{QD2} ***A_2***	τ_{QD3} ***A_3***	$\langle\tau_{QD}\rangle$*	τ_{1HT} ***B_1***
0.99 ± 0.02 ns *55.32 ± 0.91 %*	9.65 ± 0.17 ns *35.57 ± 0.68 %*	69.78 ± 1.22 ns *9.11 ± 0.26 %*	10.34 ± 0.17 ns	0.58 ± 0.06 ns *100 %*

Hole Transfer Kinetics with Various $V_6O_7^{1-}$ Equivalents		
**# Equiv. $V_6O_7^{1-}$**	**# acceptors**	τ_{nHT}#
**1**	0.20 ± 0.02	2.89 ± 0.65 ns
**2**	0.33 ± 0.03	1.76 ± 0.35 ns
**5**	0.56 ± 0.02	1.05 ± 0.15 ns
**10**	0.61 ± 0.01	0.95 ± 0.12 ns
**20**	0.57 ± 0.02	1.03 ± 0.14 ns

*Amplitude-weighted average lifetime $\langle\tau_{QD}\rangle = \frac{\sum_i A_i \tau_{QDi}}{\sum_i A_i}$. #Hole transfer lifetime ($\frac{1}{k_{nj}} = \frac{n}{k_{1HT}}$). All errors are estimated by the standard deviation of multiple measurements.

Table S4. Fitting parameters for TCSPC lifetime measurements of OA-CdSe QDs + 10 eq MS with $V_6O_7^{1-}$ clusters.

**Intrinsic Relaxation Kinetics**				**Hole Transfer Kinetics**
**τ_{QD1}**<br>***A_1***	**τ_{QD2}**<br>***A_2***	**τ_{QD3}**<br>***A_3***	**$\langle\tau_{QD}\rangle$***	**τ_{1HT}**<br>***B_1***
1.05 ± 0.02 ns<br>*53.37 ± 1.16 %*	9.76 ± 0.12 ns<br>*37.65 ± 0.91 %*	69.62 ± 1.22 ns<br>*8.98 ± 0.26 %*	10.49 ± 0.36 ns	0.70 ± 0.09 ns<br>*100 %*

**Hole Transfer Kinetics with Various $V_6O_7^{1-}$ Equivalents**		
**# Equiv. $V_6O_7^{1-}$**	**# acceptors**	**τ_{nHT}#**
**1**	0.14 ± 0.01	5.03 ± 0.96 ns
**2**	0.38 ± 0.01	1.86 ± 0.25 ns
**5**	0.65 ± 0.03	1.07 ± 0.15 ns
**10**	0.85 ± 0.02	0.83 ± 0.13 ns
**20**	0.84 ± 0.05	0.83 ± 0.07 ns

*Amplitude-weighted average lifetime $\langle\tau_{QD}\rangle = \frac{\sum_i A_i \tau_{QDi}}{\sum_i A_i}$. #Hole transfer lifetime ($\frac{1}{k_{nj}} = \frac{n}{k_{1HT}}$). All errors are estimated by the standard deviation of multiple measurements.

Table S5. Fitting parameters for TCSPC lifetime measurements of OA-CdSe QDs + 15 eq MS with $V_6O_7^{1-}$ clusters.

**Intrinsic Relaxation Kinetics**				**Hole Transfer Kinetics**
τ_{QD1} ***A_1***	τ_{QD2} ***A_2***	τ_{QD3} ***A_3***	$\langle\tau_{QD}\rangle$*	τ_{1HT} ***B_1***
1.11 ± 0.00 ns *52.41 ± 0.39 %*	9.85 ± 0.08 ns *39.11 ± 0.28 %*	69.13 ± 0.28 ns *8.48 ± 0.13 %*	10.29 ± 0.10 ns	0.83 ± 0.04 ns *100 %*

**Hole Transfer Kinetics with Various $V_6O_7^{1-}$ Equivalents**		
**# Equiv. $V_6O_7^{1-}$**	**# acceptors**	τ_{nHT}#
**1**	0.23 ± 0.03	3.62 ± 0.60 ns
**2**	0.47 ± 0.01	1.76 ± 0.13 ns
**5**	1.04 ± 0.03	0.80 ± 0.06 ns
**10**	1.32 ± 0.02	0.63 ± 0.02 ns
**20**	1.31 ± 0.03	0.63 ± 0.05 ns

*Amplitude-weighted average lifetime $\langle\tau_{QD}\rangle = \frac{\sum_i A_i \tau_{QDi}}{\sum_i A_i}$. #Hole transfer lifetime ($\frac{1}{k_{nj}} = \frac{n}{k_{1HT}}$). All errors are estimated by the standard deviation of multiple measurements.

Table S6. Fitting parameters for TCSPC lifetime measurements of OA-CdSe QDs + 20 eq MS with $V_6O_7^{1-}$ clusters.

**Intrinsic Relaxation Kinetics**				**Hole Transfer Kinetics**
τ_{QD1} ***A_1***	τ_{QD2} ***A_2***	τ_{QD3} ***A_3***	$\langle\tau_{QD}\rangle^*$	τ_{1HT} ***B_1***
1.15 ± 0.03 ns *52.69 ± 1.19 %*	9.64 ± 0.17 ns *39.85 ± 0.84 %*	67.73 ± 1.16 ns *7.46 ± 0.35 %*	9.50 ± 0.17 ns	1.00 ± 0.08 ns *100 %*

**Hole Transfer Kinetics with Various $V_6O_7^{1-}$ Equivalents**		
**# Equiv. $V_6O_7^{1-}$**	**# acceptors**	$\tau_{nHT}^{\#}$
**1**	0.26 ± 0.02	3.90 ± 0.53 ns
**2**	0.51 ± 0.01	1.97 ± 0.13 ns
**5**	1.29 ± 0.02	0.78 ± 0.07 ns
**10**	1.53 ± 0.02	0.65 ± 0.05 ns
**20**	1.95 ± 0.07	0.51 ± 0.04 ns

*Amplitude-weighted average lifetime $\langle\tau_{QD}\rangle = \frac{\sum_i A_i \tau_{QDi}}{\sum_i A_i}$. #Hole transfer lifetime ($\frac{1}{k_{nj}} = \frac{n}{k_{1HT}}$). All errors are estimated by the standard deviation of multiple measurements.

Table S7. Calculated values of θ (shaded region) across all equivalents of added MS and upon addition of $V_6O_7^{1-}$. Also shown are values for parameters θ_{max} and K_a for all equivalents of added MS.

	**Added MS**					
	**0eq**	**2eq**	**5eq**	**10eq**	**15eq**	**20eq**
**0eq $V_6O_7^{1-}$**	0%	0%	0%	0%	0%	0%
**1eq $V_6O_7^{1-}$**	0%	0.08%	0.35%	0.28%	0.51%	0.56%
**2eq $V_6O_7^{1-}$**	0%	0.18%	0.62%	0.79%	0.93%	0.94%
**5eq $V_6O_7^{1-}$**	0%	0.28%	1.23%	1.55%	2.02%	2.49%
**10eq $V_6O_7^{1-}$**	0%	0.34%	1.45%	1.96%	3.00%	3.31%
**20eq $V_6O_7^{1-}$**	0%	0.36%	1.79%	2.46%	3.47%	4.25%
**θ_{max}**	-	0.4%	2.08%	3.02%	4.37%	5.32%
**K_a (μM^{-1})**	-	0.37	0.31	0.24	0.26	0.24

Fraction accessible analysis[2]

Suppose there are two populations of fluorophores, one of which is accessible (*a*) to quenchers and the other being inaccessible or buried (*b*). The total fluorescence in the absence of quencher (F_0) is given by:

$$F_0 = F_{0a} + F_{0b} \quad (1)$$

where the subscript *0* once again refers to the fluorescence intensity in the absence of quencher. In the presence of quencher, the intensity of the accessible fraction (f_a) is decreased according to the Stern-Volmer equation, whereas the buried fraction is not quenched. Therefore, the observed intensity is given by:

$$F = \frac{F_{0a}}{1 + K_a[Q]} + F_{0b} \quad (2)$$

where K_a is the Stern-Volmer quenching constant of the accessible fraction, and [Q] is the concentration of quencher. Subtraction of equation (2) from equation (1) yields:

$$\Delta F = F_0 - F = F_{0a}\left(\frac{K_a[Q]}{1 + K_a[Q]}\right) \quad (3)$$

Inversion of equation (3) followed by division into equation (1) yields:

$$\frac{F_0}{\Delta F} = \frac{1}{f_a K_a[Q]} + \frac{1}{f_a} \quad (4)$$

where f_a is the fraction of the initial fluorescence that is accessible to quencher:

$$f_a = \frac{F_{0a}}{F_{0a} + F_{0b}} \quad (5)$$

A plot of $F_0/\Delta F$ versus $1/[Q]$ yields f_a^{-1} as the intercept and $(f_a K)^{-1}$ as the slope. The y-intercept of f_a^{-1} may be understood intuitively. The intercept represents the extrapolation to infinite quencher concentration ($1/[Q] = 0$).

References

(1) Spandl, J.; Daniel, C.; Brüdgam, I.; Hartl, H. Synthesis and structural characterization of redox-active dodecamethoxoheptaoxohexavanadium clusters. *Angew. Chem. Int. Ed.* **2003**, *42*, 1163-1166.

(2) Lakowicz, J. R. *Principles of Fluorescence Spectroscopy*, 3rd ed.; Springer: New York, 2006.

(3) Sadhu, S.; Tachiya, M.; Patra, A. A Stochastic Model for Energy Transfer from CdS Quantum Dots/Rods (Donors) to Nile Red Dye (Acceptors). *J. Phys. Chem. C* **2009**, *113*, 19488–19492.

(4) Cogan, N. M. B.; McClelland, K. P.; Peter, C. Y. M.; Carmenate Rodríguez, C.; Fertig, A. A.; Amin, M.; Brennessel, W. W.; Krauss, T. D.; Matson, E. M. Efficient Hole Transfer from CdSe Quantum Dots Enabled by Oxygen-Deficient Polyoxovanadate-Alkoxide Clusters. *Nano Lett.* **2023**, *23*, 10221–10227.

(5) Burke, R.; Cogan, N. M. B.; Oi, A.; Krauss, T. D. Recovery of Active and Efficient Photocatalytic H_2 Production for CdSe Quantum Dots. *J. Phys. Chem. C* **2018**, *122*, 14099–14106.

(6) Anderson, N. C.; Hendricks, M. P.; Choi, J. J.; Owen, J. S. Ligand Exchange and the Stoichiometry of Metal Chalcogenide Nanocrystals: Spectroscopic Observation of Facile Metal-Carboxylate Displacement and Binding. *J. Am. Chem. Soc.* **2013**, *135*, 18536–18548.

(7) Knauf, R. R.; Lennox, J. C.; Dempsey, J. L. Quantifying Ligand Exchange Reactions at CdSe Nanocrystal Surfaces. *Chem. Mater.* **2016**, *28*, 4762–4770.

(8) De Roo, J.; Yazdani, N.; Drijvers, E.; Lauria, A.; Maes, J.; Owen, J. S.; Van Driessche, I.; Niederberger, M.; Wood, V.; Martins, J. C.; Infante, I.; Hens, Z. Probing Solvent-Ligand Interactions in Colloidal Nanocrystals by the NMR Line Broadening. *Chem. Mater*. **2018**, *30*, 5485–5492.
